# Mental Health of Staff at Correctional Facilities in the United States During the COVID-19 Pandemic

**DOI:** 10.3389/fpsyt.2021.767385

**Published:** 2022-01-25

**Authors:** M. Haroon Burhanullah, Pamela Rollings-Mazza, Jeffrey Galecki, Michael Van Wert, Thomas Weber, Mansoor Malik

**Affiliations:** ^1^Department of Psychiatry and Behavioral Sciences, Johns Hopkins University School of Medicine, Baltimore, MD, United States; ^2^PrimeCare Medical, Inc., Harrisburg, PA, United States

**Keywords:** correctional health, correctional officers, resilience, burnout, mental health

## Abstract

**Background:**

Although United States (US) correctional workers (correctional officers and health care workers at correctional institutions) have experienced unprecedented stress during the COVID-19 pandemic, to date, there are no systematic data on the mental health impact of COVID-19 on correctional workers.

**Objective:**

To determine the perceived mental health burden of the COVID-19 pandemic on correctional workers and to explore the relationship between workers' mental health, social demographics, and environmental/work factors. In particular, the study sought to examine if occupational role (correctional officers vs. health care workers) or sex were associated with mental health status.

**Methods:**

This cross-sectional survey was conducted in 78 correctional sites in Pennsylvania, Maryland, West Virginia and New York from November 1 to December 1, 2020. There were 589 participants, including 103 correctional officers and 486 health care workers employed at the correctional facilities. Measurements included the Patient Health Questionnaire-9, Generalized Anxiety Disorder-7, Adult PROMIS Short Form v.1.0—Sleep Disturbance, Impact of Event Scale-Revised, Maslach Burnout Inventory 2-item, and Connor-Davidson Resilience Scale 2-item.

**Results:**

Approximately 48% of healthcare workers and 32% of correctional officers reported mild to severe depressive symptoms, 37% reported mild to severe anxiety symptoms, 47% of healthcare workers and 57% of correctional officers reported symptoms of burnout, and 50% of healthcare workers and 45% of correctional officers reported post-traumatic stress symptoms. Approximately 18% of healthcare workers and 11% of correctional officers reports mild to moderate sleep disturbance. Health care workers had significantly higher depression and sleep disturbance scores than did correctional officers, while correctional officers had significantly higher burnout scores. Female correctional workers scored significantly higher on anxiety than their male counterparts. Increased workload, workplace conflict, younger age of employees, trust in institutional isolation practices, and lower work position were associated with increased burnout. Despite experiencing high mental health burden, correctional workers showed high resilience (60%).

**Conclusion:**

We found a high level of psychological symptoms among health care workers in correctional settings, and this population may experience unique challenges, risks and protective factors relative to other health care workers outside of correctional settings. Understanding these factors is essential for developing effective interventions for correctional workers.

## Introduction

The United States (US) prison system, which holds almost 2.3 million prisoners (1), and employs more than 500,000 correctional workers (includes correctional officers and health care workers) (2), is chronically understaffed and under-resourced (3). Added to these significant challenges is the impact of the COVID-19 pandemic, which has significantly increased the work demands for correctional workers (4).

One in every five state and federal prisoners in the United States has tested positive for COVID-19, a rate more than four times higher than the general population^1^. Prisons have a high prevalence of chronic diseases and mental illness and house an increasingly aging population that is vulnerable to viral illness (5). Furthermore, poor personal hygiene contributing to virus spread, is exacerbated by such prison conditions as overcrowding, poor ventilation, close habitation, and strict control of everyday items such as soap, cleaning supplies, and hand sanitizers. Risks and vulnerabilities are borne not only by the prisoners, but also by the correctional workers, who share all the risks of the physical environment and the additional risks of inmate violence, gang activity, and uncontrolled physical contact as they move prisoners or intervene in altercations, and when performing physical examinations and medical procedures (6). Moreover, prisoners unable or unwilling to maintain personal hygiene may intentionally expose staff to body fluids (7).

Working in correctional facilities has been associated with high levels of stress and burnout (8–12). For example, correctional officers have suicide rates that are 40–100% higher than those of police officers outside of prison (3). Yet despite the enormous burden of COVID-19 on the prison system, there are no US studies examining the mental health of correctional workers during the COVID-19 pandemic.

We note with concern, that during the pandemic, health care workers in general hospital settings have increased rates of depression, anxiety, and PTSD (13–17), and we hypothesized this also to be true in the correctional community. Studying the health of correctional officers and prison health care workers during COVID-19 in a systematic way is urgently needed, to identify both the unique risk factors in vulnerable staff and to allocate scarce resources in correctional settings.

Our present study is the first to survey US correctional officers and health care workers during the COVID-19 pandemic. The primary aim of this exploratory study was to evaluate the perceived mental health of correctional workers during the COVID-19 pandemic and examine whether the mental health of correctional health care workers differed from that of correctional officers. Although both correctional officers and correctional healthcare workers work together in the challenging environments with persons who have complex needs, their duties and mandate in the correctional setting differ. Correctional officers have primarily security function while healthcare workers are primarily responsible for well-being of imprisoned populations. Correctional officers are more likely to be exposed to potentially psychologically traumatic events such as inmate violence. There are also variable and sometimes contradictory levels of organizational and peer support for these two groups. Many mental health nurses reported that they were often or always supported regarding the emotional demands of their job and consulted on changes at work, yet most correctional officers reported little or no support or consultation (18). We hypothesized that officers would report higher rates of symptoms of mental disorders, including PTSD, depression and anxiety disorders as compared to correctional health workers. The study also examined whether social factors, such as sex, and environmental/work factors, such as worker rank in their occupational hierarchy, presence of workplace conflict, and working in units with COVID-19-infected people, were associated with perceived mental health burden.

## Methods and Materials

### Study Design

This cross-sectional study was conducted at 78 correctional sites in the US. Health care workers were included from 78 sites in New York, Pennsylvania, Maryland, and West Virginia. These sites had different capacities, ranging from one to 25 health care workers. Only five of the 78 sites showed interest in surveying their correctional officers, as correctional administration was concerned about overburdening the officers due to the pandemic. Two of the sites declined later and therefore correctional officers were ultimately surveyed at the remaining three sites. The survey was administered between November 1, 2020, and December 1, 2020, at the beginning of the third peak of COVID-19 in the US, with new daily cases averaging in excess of 200,000 per day on November 21^2^.

Approval to conduct this survey was obtained from an independent IRB (Solutions IRB) and the Department of Corrections Research Review Committee, in accordance with the principles in the Declaration of Helsinki and the Common Rule. Informed consent was provided by all survey participants prior to their enrollment. Participants accessed the survey using an anonymous email link *via* Qualtrics. No personal identification information was collected. Participants could terminate the survey at any time they desired. The survey was anonymous, and confidentiality of information was assured.

### Participants

All correctional workers who were at least 18 years of age and employed as either a health care worker or correctional officer at the participating correctional sites were eligible to participate. Health care workers included doctors, nurses, social workers/counselors, nurse practitioners, physician assistants, medical assistants and patient care technicians. Correctional officers included correctional officers, correctional supervisors (captains, lieutenants), and correctional administrators (assistant wardens, deputy wardens, and wardens).

The target sample size of participants was determined using the formula (19) *N* = Zα^2^ P(1 – P)/d^2^, in which α = 0.05 and Zα = 1.96, and the estimated acceptable margin of error for proportion d was 0.1. The proportion of health care workers with psychological comorbidities was estimated at 35%, based on a previous systematic review (17), resulting in an estimate sample size of 88 in each group. To allow for subgroup analyses, we amplified the sample size by 50%, with a goal of at least 132 completed questionnaires from each of the two groups (thus at least 264 completed questionnaires in total). Using a convenience sample methodology, we emailed the survey to 200 correctional officers from three sites and 103 of them completed the survey. Likewise, 1,500 health care workers from 78 sites were sent the survey and 486 completed it.

### Main Outcome Measures

For all participants, we assessed mood, anxiety, sleep, trauma, burnout, and resilience using validated measurement tools as follows: (i) depression, nine-item Patient Health Questionnaire (20) (PHQ-9, range 0–29); (ii) anxiety, seven-item Generalized Anxiety Disorder (21) scale (GAD-7, range 0–21); (iii) sleep disturbance, four-item PROMIS Sleep Disturbance Index short form (22–24) (PSDI, range 32–73); and (iv) post-traumatic stress, 22-item Impact of Event Scale–Revised (25) (IES-R, range 0–88). To measure burnout, and resilience, we used modified versions of the Maslach Burnout Inventory (MBI-2) (26, 27) and the Connor-Davidson Resilience Scale (CD-RISC-2) (28), respectively.

The total scores of these measurement tools were interpreted as follows:

PHQ-9: mild (5–9), moderate (10–14), moderately severe (15–19), and severe (20–27) depression.

GAD-7: normal (0–4), mild (5–9), moderate (10–14), and severe (15–21) anxiety.

Adult PROMIS Sleep Disturbance: raw scores are converted to t-scores which range from 32 to 73.3: 32–54: within normal limits, 55–60: mild, 61–70: moderate, 71–73.3: severe sleep disturbance.

IES-R: normal (0–8), mild (9–25), moderate (26–43), and severe (44–88) distress.

MBI-2: low burnout: not answering once a week or more on either question vs. high burnout: answering once a week or more on either question.

CD-RISC-2: lower resiliency: < 6 vs. higher resiliency: 6<.

All participants had the opportunity to answer a survey comprising of six questions/statements related to COVID-19. These have not been psychometrically tested, although a similar questionnaire has previously been used in SARS outbreak (29). Given the unprecedented nature of COVID pandemic, there was no opportunity to validate the survey and therefore it has only face validity. The six questions/statements with assigned variable names in parenthesis were: “Do you think the current practices and protective equipment at your institution protect you from contracting the COVID-19 infection?” (Isolation Practices), “I have been afraid of falling ill of COVID-19.” (COVID-19 Fear), “I think that I have had an increase in my workload such as increase in intensity of work, or number of patients etc.” (Increased Workload), “I think that there is more conflict amongst colleagues at work.” (Work Conflict), “Have you worked at a unit/location dedicated for patients/inmates with confirmed COVID-19?” (COVID-19 Unit Infection), and there were two questions related to self-isolation. “Have you been quarantined (strict self-isolation for several days) due to your contact or a personal diagnosis of COVID-19?” (Quarantine) and “Have you been in contact with anyone who has been diagnosed with COVID-19 infection?”

Demographic data were self-reported by the participants, including work position (health care worker vs. correctional officer), sex (male or female), age (18–25, 26–35, 36–49, 50–56, or >56 years), living situation (lives alone or not), and presence or absence of a chronic medical condition.

### Statistical Analysis

IBM SPSS 27.0.1.0 (Corp., 2020) was used for all data preparation and analysis. Significance was assessed at the 0.05 level unless otherwise noted. To examine the differences between health care workers and correctional officers on each of the nine mental health outcome measures, independent-samples *t*-tests and a Mann Whitney *U*-test were conducted. Using the Bonferroni adjustment (30) to aid in controlling the family-wise error rate from multiple comparisons, an alpha of 0.05 divided by the nine comparisons resulted in an adjusted alpha of 0.005 (e.g., 0.05/9 = 0.005) as the threshold for significance. The Mann Whitney *U* effect size was calculated based on Rosenthal's (31) method for converting a *z*-score to an effect size estimate (e.g., dividing *z* by the square root of *N*) (31). Cohen's *d* was calculated for each independent samples *t*-test with the suggestion by Cohen (32) that *d* = 0.20 is a small effect, *d* = 0.50 is a medium effect, and *d* = 0.80 is a large effect (32).

A series of multiple linear regressions were conducted with predictor variables on each of the mental health outcome measures. The assumption of normally distributed errors was examined by visual inspection of residuals histograms and plots (33, 34). To assess autocorrelation the Durban-Watson statistic was calculated with values <1 or >3 indicating departure from this assumption (35). Multicollinearity was assessed by examining the correlation matrix for predictor variables that correlate very highly which is problematic in the interpretation of the models (36) and by examining the variance inflation factor (VIF) and tolerance (the reciprocal of VIF) where VIF > 10, or an average VIF markedly larger than 1 (37), or tolerance below 0.2 being a cause for concern. These regression models yielded unstandardized (*B*) and standardized (β) coefficients (36). Unstandardized coefficients (*B*) indicated the amount that a mental health outcome measure score changed as a given predictor variable changed by one unit, keeping all other predictor variables constant. To facilitate the comparison of coefficient sizes across outcome measure scores with different scales, the standardized coefficients (β) converted scores to standard deviations. For instance, a β value of 2.15 indicates that a change of one standard deviation in a predictor corresponds to an increase of 2.15 standard deviations in the outcome measure scores.

For multiple linear regression analyses, the following variables were coded as 0 = No and 1 = Yes: COVID-19 fear, increased workload, work conflict, COVID-19-unit infection, quarantined, self-isolation, chronic medical (condition), and lives alone. Sex was coded as 0 = Male and 1 = Female, and work position as 0 = health care worker and 1 = correctional officer. Age and isolation practices were coded along an ordinal scale but treated as continuous for analysis.

## Results

Respondent demographic characteristics are presented in Table 1. Most of the respondents were female (76.3%), ages 25–50 (70%) and white/Caucasian (86.4%). Most of the respondents (82.5%) were healthcare workers and only 17.5% were correctional officers.

**Table 1:** Demographic characteristics of participants (*N* = 589).

**Characteristic**	***N***	**%**
Age at time of survey
18–24	34	5.9
25–34	173	30.0
35–49	231	40.0
50–56	139	24.1
Sex
Female	447	76.3
Male	139	23.7
Race
White or Caucasian	509	86.4
Black or African American	42	7.1
Hispanic or Latino	13	2.2
Asian or Asian American	4	0.7
American Indian or Alaska Native	2	0.3
Worker position
Health care workers	486	82.5
Correctional officers	103	17.5
Living status
Not alone	540	91.7
Alone	49	8.3
Chronic medical conditions
No	381	64.7
Yes	199	33.8

The survey was emailed to 1,700 correctional workers, and out of these, 900 opened their email (60%). Of those 900, 589 (486 health care workers and 103 correctional officers) completed the survey (65%). Descriptive statistics comparing health care worker and correctional officer mental health are presented in Table 2.

**Table 2:** Categorized scores on mental health measures for health care workers and correctional officers.

**Scale**	**Healthcare workers**	**Correctional officers**
	***N* = 483**	***N* = 103**
PHQ-9
None (0–4)	249 (51.55)	70 (67.96)
Mild (5–9)	131 (27.12)	24 (23.30)
Moderate (10–14)	69 (14.29)	6 (5.82)
Moderately severe (15–19)	28 (6.80)	3 (2.91)
Severe (20–27)	6 (1.24)	0 (0.00)
GAD-7
Minimal anxiety (0–4)	228 (47.20)	57 (55.34)
Mild anxiety (5–9)	125 (25.88)	29 (28.15)
Moderate anxiety (10–14)	62 (12.84)	10 (9.70)
Severe anxiety (15–21)	0 (0.00)	0 (0.00)
MBI-2
Low	254 (52.58)	43 (41.75)
High	228 (47.20)	59 (57.28)
PROMIS sleep
Normal (32–54)	394 (81.57)	89 (86.41)
Mild (55–60)	84 (17.39)	11 (10.68)
Moderate (61–70)	1 (0.02)	1 (0.97)
Severe (71–73)	0 (0.00)	0 (0.0)
IES-R total
Normal (0–8)	209 (43.27)	46 (44.66)
Mild (9–28)	140 (28.99)	28 (27.18)
Moderate (26–43)	60 (12.42)	14 (13.59)
Severe (44–88)	38 (7.87)	5 (4.85)
CD-RISC-2
Low (<6)	214 (44.30)	36 (34.95)
High (>6)	269 (55.69)	65 (63.11)

*Numbers in parenthesis are percentages*.

### Comparison of Health Care Worker and Correctional Officer Mental Health

Independent sample *t*-tests were conducted on the eight continuous measures. These *t*-tests indicated statistically significant differences (adjusted 0.005 level) between health care workers and correctional officers (see Table 3). Specifically, health care worker mean PHQ-9 depression score (*M* = 5.74, *SD* = 5.15) was higher than that of correctional officers (*M* = 3.96, *SD* = 3.86) [*t*_(182.70)_ = 3.94, *p* < 0.001]. Health care worker mean GAD-7 anxiety score (*M* = 6.24, *SD* = 5.91) was also higher than that of correctional officers (*M* = 4.66, *SD* = 4.48) [*t*_(185.56)_ = 3.05, *p* = 0.003]. Finally, health care worker mean PROMIS sleep disturbance score (*M* = 10.98 *SD* = 1.81) was higher than that of correctional officers (*M* = 10.37, *SD* = 1.79) [*t*_(578)_ = 3.06, *p* = 0.002]. In contrast, correctional officer mean MBI-2 burnout score (*M* = 3.51, *SD* = 2.99) was higher than that of health care workers (*M* = 4.53, *SD* = 3.15) [*t*_(579)_ = −3.09, *p* = 0.002]. No statistically significant differences were found between the two groups for IES-R total score, IES - Avoidance, IES - Hyperarousal, or IES - Intrusion scores. Due to its ordinal response format, a Mann-Whitney *U*-test was conducted on the CD-RISC-2 resilience scale and found that health care workers (*n* = 483, *Mdn* = 7.00) did not significantly differ from correctional officers (*n* = 101, *Mdn* = 7.00), *U* = 21986.50, *z* = −1.63, *p* = 0.10, *r* = −0.07.

**Table 3:** Differences in mental health measures between health care workers and correctional officers.

	**Health care workers**			**Correctional officers**							
**Measure**	***N***	**Mean**	***SD***	***N***	**Mean**	***SD***	***df***	***t***	***p***	**95% Confidence interval of the difference**	**Cohen's *d***
PHQ-9	472	5.74	5.15	100	3.96	3.86	182.70	3.94	**<0.001**	[0.89, 2.67]	0.36
GAD-7	473	6.24	5.91	102	4.66	4.48	185.56	3.05	**0.003**	[0.56, 2.61]	0.28
MBI-2	481	3.51	2.99	100	4.53	3.15	579	−3.09	**0.002**	[−1.67, −0.37]	0.34
PROMIS sleep	479	10.98	1.81	101	10.37	1.79	578	3.06	**0.002**	[0.60, 0.20]	0.36
IES-R											
Total	447	15.42	16.14	93	14.08	15.28	538	0.73	0.46	[−2.24, 4.92]	0.08
Avoidance	459	5.35	5.88	98	5.84	6.79	555	−0.72	0.47	[−1.81, 0.84]	0.08
Hyperarousal	474	4.31	4.88	100	3.36	4.22	572	1.81	0.07	[−0.08, 1.98]	0.20
Intrusion	468	5.96	6.42	101	4.29	5.83	567	2.41	0.02	[0.31, 3.03]	0.26

*Due to multiple comparisons, alpha was adjusted to 0.005, with bold values indicating significance at this adjusted alpha level*.

### Mental Health Severity and Associated Factors

Nine multiple linear regression models were conducted to evaluate the association between mental health domains and a variety of variables. Visual inspection of the residual histogram and plot combined with sample sizes indicated the assumption of normally distributed errors was not violated to an extent that would cause concern. Durban-Watson values were not <1 or >3 and thus the autocorrelation was determined to not be an issue. Multicollinearity was assessed by examining correlation matrices (Appendix A), VIF, and tolerance. Based on previously set guidance, multicollinearity was not thought to be operating to a concerning level in the models that follow.

Workers who believed that institutional isolation practices were protective (β = 0.17, *p* < 0.001), felt afraid of falling ill to COVID-19 (β = 0.14, *p* < 0.001), experienced an increased workload (β = 0.18, *p* < 0.001), experienced more work conflict (β = 0.15, *p* < 0.001), were of younger age (β = −0.10, *p* = 0.02) and had chronic medical conditions (β = 0.44, *p* < 0.05) scored higher on the PHQ-9. Working in a unit with COVID-19 infection, quarantine status, self-isolation status, living status, sex, and work position were not significant predictors. This regression model [*R*^2^ = 0.23, *F*_(12, 503)_ = 12.60, *p* < 0.001] accounted for 23% of the variation in PHQ-9 scores (see Table 4).

**Table 4:** Multiple linear regression analysis for worker variables predicting PHQ-9 depression score.

**Variable**	***B***	***SE B***	**95% CI**	**β**	***t***	***p***
Constant	1.91	1.07	[−0.20, 4.01]		1.78	0.08
Isolation practices^a^	0.60	0.15	[0.30, 0.89]	0.17	3.98	**<0.001**
COVID-19 fear^b^	1.44	0.43	[0.60, 2.27]	0.14	3.37	**<0.001**
Increased workload^b^	1.88	0.44	[1.02, 2.74]	0.18	4.30	**<0.001**
Work conflict^b^	1.53	0.44	[0.67, 2.38]	0.15	3.50	**<0.001**
COVID-19 unit infection^b^	0.18	0.42	[−0.64, 1.01]	0.02	0.44	0.66
Quarantined^b^	0.31	0.54	[−0.75, 1.37]	0.03	0.58	0.56
Age^c^	−0.58	0.24	[−1.05, −0.10]	−0.10	−2.40	**0.02**
Sex^d^	0.52	0.56	[−0.59, 1.62]	0.04	0.92	0.36
Self-isolation^b^	0.88	0.61	[−0.32, 2.09]	0.06	1.45	0.15
Chronic medical condition^b^	0.95	0.44	[0.08, 0.08]	0.09	2.15	**0.03**
Lives alone^b^	1.42	0.79	[−0.14, 2.97]	0.08	1.79	0.07
Work position^e^	0.03	0.64	[−1.22, 1.28]	0.00	0.04	0.97

*R^2^ =0.23 (N = 515, p < 0.001). CI = confidence interval for B. Values in bold indicate significance of p < 0.05. Isolation Practices and Age were treated as continuous variables*.

a *1 = Definitely yes, 2 = Probably yes, 3 = Might or might not, 4 = Probably not, 5 = Definitely not*.

b *0 = No, 1 = Yes*.

c *1 = 18–24, 2 = 25–34, 3 = 35–49, and 4 = 50–65*.

d *0 = Male, 1 = Female*.

e *0 = Health Care Worker, 1 = Correctional Officer*.

Mean GAD-7 anxiety score was higher among workers who believed that isolation practices were protective (β = 0.10, *p* = 0.01), felt afraid of falling ill to COVID-19 (β = 0.20, *p* < 0.001), experienced increased workload (β = 0.17, *p* < 0.001), more work conflict (β = 0.17, *p* < 0.001), were younger (β = −0.20, *p* < 0.001), and female (β = 0.10, *p* = 0.04). Working on a unit with COVID-19 infection, self-isolation status, chronic medical condition status, living status and work position were not significant predictors. The model [*R*^2^ = 0.26, *F*_(12, 508)_ = 15.01, *p* < 0.001] accounted for 26% of the variation in GAD-7 scores (see Table 5).

**Table 5:** Multiple linear regression analysis for worker variables predicting GAD-7 anxiety score.

**Variable**	***B***	***SE B***	**95% CI**	**β**	***t***	***p***
Constant	3.36	1.20	[1.01, 5.71]		2.80	**0.005**
Isolation practices^a^	0.42	0.17	[0.09, 0.75]	0.10	2.51	**0.01**
COVID-19 fear^b^	2.27	0.47	[1.34, 3.20]	0.20	4.80	**<0.001**
Increased workload^b^	2.02	0.49	[1.07, 2.98]	0.17	4.16	**<0.001**
Work conflict^b^	1.94	0.48	[0.90, 2.89]	0.17	4.01	**<0.001**
COVID-19 unit infection^b^	0.19	0.47	[−0.72, 1.10]	0.02	0.41	0.68
Quarantined^b^	0.96	0.59	[−0.21, 2.12]	0.07	1.62	0.11
Age^c^	−1.21	0.27	[−1.73, −0.68]	−0.18	−4.50	**<0.001**
Sex^d^	1.31	0.62	[0.09, 2.53]	0.10	2.11	**0.04**
Self-isolation^b^	0.57	0.66	[−0.73, 1.87]	0.04	0.86	0.39
Chronic medical condition^b^	0.68	0.49	[−0.28, 1.64]	0.06	1.40	0.16
Lives alone^b^	0.16	0.86	[−1.54, 1.85]	0.01	0.18	0.86
Work position^e^	0.89	0.70	[−0.49, 2.27]	0.06	1.27	0.21

*R^2^ =0.26 (N = 515, p < 0.001). CI = confidence interval for B. Values in bold indicate significance of p < 0.05. Isolation Practices and Age were treated as continuous variables*.

a *1 = Definitely yes, 2 = Probably yes, 3 = Might or might not, 4 = Probably not, 5 = Definitely not*.

b *0 = No, 1 = Yes*.

c *1 = 18–24, 2 = 25–34, 3 = 35–49, and 4 = 50–65*.

d *0 = Male, 1 = Female*.

e *0 = Health Care Worker, 1 = Correctional Officer*.

Mean MBI-2 burnout score was higher among workers who believed that isolation practices were protective (β = 0.25, *p* < 0.001), experienced increased workload (β = 0.21, *p* < 0.001), more work conflict (β = 0.25, *p* < 0.001), were younger (β = −0.11, *p* = 0.004), and had lower work position (β = 0.26, *p* < 0.001). COVID-19 fear, working on a COVID-19 unit, quarantine status, sex, self-isolation status, chronic medical condition status, and living status were not significant predictors. The model [*R*^2^ = 0.32, *F*_(12, 513)_ = 20.16, *p* < 0.001] accounted for 32% of the variation in MBI-2 scores (see Table 6). A linear regression predicting PROMIS sleep disturbance (see Table 7) resulted in no significant predictor variables [*R*^2^ =0.04, *F*_(12, 511)_ = 1.65, *p* = 0.07].

**Table 6:** Multiple linear regression analysis for worker variables predicting MBI-2 burnout score.

**Variable**	***B***	***SE B***	**95% CI**	**β**	***t***	***p***
Constant	0.75	0.61	[−0.44, 1.94]		1.24	0.22
Isolation practices^a^	0.53	0.08	[0.36, 0.70]	0.25	6.28	**<0.001**
COVID-19 fear^b^	0.38	0.24	[−0.09, 0.85]	0.06	1.60	0.11
Increased workload^b^	1.32	0.25	[0.84, 1.80]	0.21	5.37	**<0.001**
Work conflict^b^	1.53	0.24	[1.05, 2.01]	0.25	6.29	**<0.001**
COVID-19 unit infection^b^	0.27	0.23	[−0.19, 0.73]	0.04	1.15	0.25
Quarantined^b^	0.53	0.30	[−0.05, 1.11]	0.07	1.79	0.08
Age^c^	−0.39	0.14	[−0.65, −0.12]	−0.11	−2.86	**0.004**
Sex^d^	0.36	0.31	[−0.25, 0.98]	0.05	1.16	0.25
Self-isolation^b^	−0.50	0.33	[−1.15, 0.16]	−0.06	−1.48	0.14
Chronic medical condition^b^	0.29	0.25	[−0.19, 0.77]	0.05	1.18	0.24
Lives alone^b^	0.53	0.44	[−0.32, 1.39]	0.06	1.22	0.22
Work position^e^	2.09	0.36	[1.39, 2.80]	0.26	5.86	**<0.001**

*R^2^ = 0.32 (N = 525, p < 0.001). CI = confidence interval for B. Values in bold indicate significance of p < 0.05. Isolation Practices and Age were treated as continuous variables*.

a *1 = Definitely yes, 2 = Probably yes, 3 = Might or might not, 4 = Probably not, 5 = Definitely not*.

b *0 = No, 1 = Yes*.

c *1 = 18–24, 2 = 25–34, 3 = 35–49, and 4 = 50–65*.

d *0 = Male, 1 = Female*.

e *0 = Health Care Worker, 1 = Correctional Officer*.

**Table 7:** Multiple linear regression analysis for worker variables predicting PROMIS sleep disturbance score.

**Variable**	***B***	***SE B***	**95% CI**	**β**	***t***	***p***
Constant	10.68	0.44	[9.83, 11.54]		24.57	**<0.001**
Isolation practices^a^	0.08	0.06	[−0.04, 0.19]	0.06	1.26	0.21
COVID-19 fear^b^	0.15	0.04	[−0.19, 0.48]	0.04	0.85	0.39
Increased workload^b^	−0.15	0.18	[−0.49, 0.20]	0.40	−0.85	0.40
Work conflict^b^	−0.08	0.17	[−0.42, 0.26]	−0.02	−0.47	0.64
COVID-19 unit infection^b^	0.18	0.17	[−0.15, 0.51]	0.05	1.06	0.29
Quarantined^b^	−0.04	0.21	[−0.45, 0.38]	−0.01	−0.17	0.87
Age^c^	−0.09	−0.04	[−0.28, 0.10]	−0.04	−0.94	0.35
Sex^d^	0.26	0.23	[−0.18, 0.71]	0.06	1.17	0.24
Self-isolation^b^	0.03	0.24	[−0.43, 0.50]	0.01	0.14	0.89
Chronic medical condition^b^	0.22	0.18	[−0.13, 0.56]	0.06	1.25	0.21
Lives alone^b^	−0.24	0.32	[−0.87, 0.38]	−0.03	−0.77	0.45
Work position^e^	−0.33	0.26	[−0.84, 0.17]	−0.07	−1.30	0.20

*R^2^ =0.19 (N = 523, p = 0.07). CI = confidence interval for B. Values in bold indicate significance of p < 0.05. Isolation Practices and Age were treated as continuous variables*.

a *1 = Definitely yes, 2 = Probably yes, 3 = Might or might not, 4 = Probably not, 5 = Definitely not*.

b *0 = No, 1 = Yes*.

c *1 = 18–24, 2 = 25–34, 3 = 35–49, and 4 = 50–65*.

d *0 = Male, 1 = Female*.

e *0 = Health Care Worker, 1 = Correctional Officer*.

Mean IES-R total score was higher among workers who believed that isolation practices were protective (β = 0.13, *p* = 0.003), reported fear of COVID-19 infection (β = 0.20, *p* < 0.001), experienced increased workload (β = 0.18, *p* < 0.001), more work conflict (β = 0.15, *p* < 0.001), had self-isolated (β = 0.11, *p* = 0.01), had a chronic medical condition (β = 0.09, *p* = 0.03), and had lower work position (β = 0.10, *p* = 0.04). Working on a COVID-19 unit, quarantine status, age, sex, and living status were not significant predictors in the model. The regression model [*R*^2^ = 0.25, *F*_(12, 480)_ = 13.25, *p* < 0.001] accounted for 25% of the variation in IES-R total scores (see Table 8).

**Table 8:** Multiple linear regression analysis for worker variables predicting IES-R posttraumatic stress total score.

**Variable**	***B***	***SE B***	**95% CI**	**β**	***t***	***p***
Constant	1.18	3.48	[−5.65, 8.01]		0.34	0.74
Isolation practices^a^	1.44	0.49	[0.48, 2.40]	0.13	2.96	**0.003**
COVID-19 fear^b^	6.37	1.37	[3.68, 9.07]	0.20	4.65	**<0.001**
Increased workload^b^	5.77	1.43	[2.97, 8.58]	0.18	4.05	**<0.001**
Work conflict^b^	4.81	1.41	[2.04, 7.58]	0.15	3.41	**<0.001**
COVID-19 unit infection^b^	2.04	1.36	[−0.63, 4.71]	0.06	1.50	0.13
Quarantined^b^	1.51	1.74	[−1.91, 4.94]	0.39	0.87	0.39
Age^c^	−1.28	0.77	[−2.80, 0.24]	−0.07	−1.66	0.10
Sex^d^	1.73	1.86	[−1.92, 5.40]	0.05	0.93	0.25
Self-isolation^b^	4.97	1.94	[1.17, 8.78]	0.11	2.57	**0.01**
Chronic medical condition^b^	3.12	1.41	[0.35, 5.90]	0.09	2.21	**0.03**
Lives alone^b^	−1.11	2.47	[−5.96, 3.73]	−0.02	−0.45	0.65
Work position^e^	4.19	2.07	[0.13, 8.24]	0.10	2.03	**0.04**

*R^2^ =0.25 (N = 492, p < 0.001). CI = confidence interval for B. Values in bold indicate significance of p < 0.05. Isolation Practices and Age were treated as continuous variables*.

a *1 = Definitely yes, 2 = Probably yes, 3 = Might or might not, 4 = Probably not, 5 = Definitely not*.

b *0 = No, 1 = Yes*.

c *1 = 18-24, 2 = 25–34, 3 = 35–49, and 4 = 50–65*.

d *0 = Male, 1 = Female*.

e *0 = Health Care Worker, 1 = Correctional Officer*.

Mean IES-R—Avoidance score was higher among workers who believed that isolation practices were protective (β = 0.13, *p* = 0.002), were afraid of COVID-19 infection (β = 0.16, *p* < 0.001), experienced increased workload (β = 0.14, *p* = 0.001), more work conflict (β = 0.55, *p* = 0.002), had a chronic medical condition (β = 0.18, *p* = 0.05), and had lower work position (β = 0.14, p = 0.006). Working in a COVID-19 unit, quarantine status, age, sex, self-isolation status, and living status were not significant predictors. The model [*R*^2^ = 0.19, *F*_(12, 495)_ = 9.61, *p* < 0.001] accounted for 19% of the variance in IES-R—Avoidance scores (see Table 9).

**Table 9:** Multiple linear regression analysis for worker variables predicting IES-R posttraumatic stress—avoidance score.

**Variable**	***B***	***SE B***	**95% CI**	**β**	***t***	***p***
Constant	1.37	1.35	[−1.28, 4.02]		1.01	0.31
Isolation practices^a^	0.58	0.19	[0.21, 0.95]	0.13	3.06	**0.002**
COVID-19 fear^b^	1.97	0.54	[0.92, 3.02]	0.16	3.69	**<0.001**
Increased workload^b^	1.78	0.56	[0.69, 2.87]	0.14	3.20	**0.001**
Work conflict^b^	1.70	0.55	[0.62, 2.78]	0.55	3.10	**0.002**
COVID-19 unit infection^b^	0.87	0.53	[−0.17, 1.90]	0.07	1.64	0.10
Quarantined^b^	0.09	0.68	[−1.25, 1.43]	0.01	0.13	0.90
Age^c^	−0.55	0.30	[−1.14, 0.05]	−0.08	−1.81	0.07
Sex	−0.05	0.71	[−1.45, 1.35]	0.00	−0.07	0.94
Self-isolation^b^	1.33	0.76	[−0.16, 2.83]	0.08	1.75	0.08
Chronic medical condition^b^	1.09	0.55	[0.01, 2.18]	0.08	1.99	**0.05**
Lives alone^b^	−1.01	0.97	[−2.92, 0.91]	−0.04	−1.04	0.30
Work position^e^	2.22	0.80	[0.65, 3.79]	0.14	2.78	**0.006**

*R^2^ =0.0.19 (N = 507, p < 0.001). CI = confidence interval for B. Values in bold indicate significance of p < 0.05. Isolation Practices and Age were treated as continuous variables*.

a *1 = Definitely yes, 2 = Probably yes, 3 = Might or might not, 4 = Probably not, 5 = Definitely not*.

b *0 = No, 1 = Yes*.

c *1 = 18–24, 2 = 25–34, 3 = 35–49, and 4 = 50–65*.

*^d^0 = Male, 1 = Female*.

e *0 = Health Care Worker, 1 = Correctional Officer*.

Mean IES-R—Hyperarousal score was higher among workers who believed that isolation practices were protective (β = 0.11, *p* = 0.009), were afraid of COVID-19 infection (β = 0.19, *p* < 0.001), experienced an increased workload (β = 0.16, *p* < 0.001), more work conflict (β = 0.16, *p* < 0.001), and had a chronic medical condition (β = 0.11, *p* = 0.01*)*. Quarantine status, age, sex, self-isolation status, living status, and work position were not significant predictors. The model [*R*^2^ = 0.22, *F*_(12, 506)_, *p* < 0.001] explained 22% of the variance in IES-R—Hyperarousal scores (see Table 10).

**Table 10:** Multiple linear regression analysis for worker variables predicting IES-R posttraumatic stress—hyperarousal score.

**Variable**	***B***	***SE B***	**95% CI**	**β**	***t***	***p***
Constant	0.10	1.03	[−1.93, 2.13]		0.10	0.92
Isolation practices^a^	0.38	0.14	[0.08, 0.66]	0.11	2.64	**0.009**
COVID-19 fear^b^	1.90	0.41	[1.09, 2.70]	0.19	4.65	**<0.001**
Increased workload^b^	1.60	0.42	[0.78, 2.43]	0.16	3.81	**<0.001**
Work conflict^b^	1.53	0.42	[0.71, 2.35]	0.16	3.67	**<0.001**
COVID-19 unit infection^b^	0.35	0.40	[−0.44, 1.14]	0.04	0.86	0.39
Quarantined^b^	0.24	0.51	[−0.76, 1.24]	0.02	0.48	0.64
Age^c^	−0.36	0.23	[−0.81, 0.09]	−0.06	−1.56	0.12
Sex^d^	0.83	0.54	[−0.23, 1.90]	0.07	1.54	0.13
Self-isolation^b^	0.99	0.57	[−0.14, 2.11]	0.07	1.72	0.09
Chronic medical condition^b^	1.08	0.42	[0.26, 1.91]	0.11	2.58	**0.01**
Lives alone^b^	−0.38	−0.02	[−1.85, 1.09]	−0.02	−0.51	0.61
Work position^e^	0.97	0.61	[−0.24, 2.17]	0.08	1.58	0.12

*R^2^ =0.0.22 (N = 518, p < 0.001). CI = confidence interval for B. Values in bold indicate significance of p < 0.05. Isolation Practices and Age were treated as continuous variables*.

a *1 = Definitely yes, 2 = Probably yes, 3 = Might or might not, 4 = Probably not, 5 = Definitely not*.

b *0 = No, 1 = Yes*.

c *1 = 18–24, 2 = 25–34, 3 = 35–49, and 4 = 50–65*.

d *0 = Male, 1 = Female*.

e *0 = Health Care Worker, 1 = Correctional Officer*.

Mean IES-R—Intrusion score was higher among workers who believed that isolation practices were protective (β = 0.11, *p* = 0.007), were afraid of COVID-19 infection (β = 0.22, *p* < 0.001), experienced an increased workload (β = 0.16, *p* < 0.001), more work conflict (β = 0.14, *p* = 0.002), and had self-isolated (β = 0.12, *p* = 0.006). Working in a COVID-19 unit, quarantine status, age, sex, chronic medical condition status, living status, and work position were not statistically significant. The model [*R*^2^ = 0.24, *F*_(12, 503)_, *p* < 0.001] accounts for 24% of the variance in IES-R—Intrusion score (see Table 11).

**Table 11:** Multiple linear regression analysis for worker variables predicting IES-R posttraumatic stress—intrusion score.

**Variable**	***B***	***SE B***	**95% CI**	**β**	***t***	***p***
Constant	0.31	1.35	[−2.35, 2.97]		0.23	0.82
Isolation practices^a^	0.51	0.51	[0.14, 0.89]	0.11	2.71	**0.007**
COVID-19 fear^b^	2.79	0.53	[1.75, 3.84]	0.22	5.24	**<0.001**
Increased workload^b^	2.06	0.55	[0.97, 3.15]	0.16	3.72	**<0.001**
Work conflict^b^	1.74	0.55	[0.66, 2.82]	0.14	3.18	**0.002**
COVID-19 unit infection^b^	0.54	0.53	[−0.49, 1.58]	0.04	1.03	0.30
Quarantined^b^	1.10	0.67	[−0.22, 2.42]	0.07	1.64	0.10
Age^c^	−0.34	0.30	[−0.93, 0.26]	−0.05	−1.11	0.27
Sex^d^	0.54	0.72	[−0.87, 1.96]	0.04	0.75	0.45
Self-isolation^b^	2.10	0.76	[0.61, 3.58]	0.12	2.77	**0.006**
Chronic medical condition^b^	0.82	0.55	[−0.27, 1.90]	0.06	1.48	0.14
Lives alone^b^	0.21	0.98	[−1.72, 2.13]	0.01	0.21	0.83
Work position^e^	0.33	0.80	[−1.24, 1.90]	0.02	0.41	0.68

*R^2^ = 0.0.24 (N = 515, p = < 0.001). CI = confidence interval for B. Values in bold indicate significance of p < 0.05. Isolation Practices and Age were treated as continuous variables*.

a *1 = Definitely yes, 2 = Probably yes, 3 = Might or might not, 4 = Probably not, 5 = Definitely not*.

b *0 = No, 1 = Yes*.

c *1 = 18–24, 2 = 25–34, 3 = 35–49, and 4 = 50–65*.

d *0 = Male, 1 = Female*.

e *0 = Health Care Worker, 1 = Correctional Officer*.

Mean CD-RISC-2 resilience score was lower among workers who feared COVID-19 infection (β = −0.21, *p* < 0.001) and females (β = −0.14, *p* = 0.008). Belief in isolation practices, experience of work conflict, working in a COVID-19 unit, quarantine status, age, chronic medical condition status, living status, and work position were not statistically significant predictors in the model. This model accounted for 10% of the variance [*R*^2^ = 0.10, *F*_(12, 514)_, *p* > 0.001] in CD-RISC-2 scores (see Table 12).

**Table 12:** Multiple linear regression analysis for worker variables predicting CD-RISC-2 resilience score.

**Variable**	***B***	***SE B***	**95% CI**	**β**	***t***	***p***
Constant	7.50	0.36	[6.80, 8.20]		20.98	**<0.001**
Isolation practices^a^	−0.11	0.05	[−0.21, −0.01]	−0.10	−2.25	0.03
COVID-19 fear^b^	−0.67	0.14	[−0.95, 0.39]	−0.21	−4.76	**<0.001**
Increased workload^b^	0.01	0.15	[−0.28, 0.30]	0.00	0.06	0.95
Work conflict^b^	−0.01	0.14	[−0.30, 0.27]	0.14	−0.10	0.92
COVID-19 unit infection^b^	0.12	0.14	[−0.16, 0.39]	0.04	0.84	0.41
Quarantined^b^	−0.32	0.18	[−0.66, 0.03]	−0.08	−1.80	0.07
Age^c^	−0.06	0.08	[−0.10, 0.22]	0.03	0.76	0.45
Sex^d^	−0.50	0.19	[−0.86, −0.13]	−0.14	−2.68	**0.008**
Self-isolation^b^	0.29	0.20	[−0.11, 0.68]	0.07	1.44	0.15
Chronic medical condition^b^	−0.12	0.15	[−0.41, 0.16]	−0.04	−0.83	0.41
Lives alone^b^	−0.43	0.26	[−0.94, 0.09]	0.26	−1.63	0.10
Work position^e^	−0.23	0.21	[−0.64, 0.19]	−0.06	−1.10	0.28

*R^2^ =0.10 (N = 526, p = < 0.001). CI = confidence interval for B. Values in bold indicate significance of p < 0.05. Isolation Practices and Age were treated as continuous variables*.

a *1 = Definitely yes, 2 = Probably yes, 3 = Might or might not, 4 = Probably not, 5 = Definitely not*.

b *0 = No, 1 = Yes*.

c *1 = 18–24, 2 = 25–34, 3 = 35–49, and 4 = 50–65*.

d *0 = Male, 1 = Female*.

e *0 = Health Care Worker, 1 = Correctional Officer*.

## Discussion

In this cross-sectional survey of 589 correctional workers, we found a high prevalence of psychological symptoms. Approximately 48% of healthcare workers and 32% of correctional officers reported mild to severe depressive symptoms, 37% reported mild to severe anxiety symptoms, 47% of healthcare workers and 57% of correctional officers reported symptoms of burnout, and 50% of healthcare workers and 45 of correctional officers reported post-traumatic stress symptoms. Although there are no previous studies for a direct comparison, these rates are higher than those reported in most of the studies on frontline hospital health care workers (13, 14).

For example, a metanalysis of 86 studies of frontline healthcare workers carried out during H1N1, Ebola, MERS, and COVID-19 found significantly lower prevalence of depression 25.72%, (95% CI 18.34–33.86), anxiety 25.36% (95% CI 17.90–33.64), posttraumatic stress disorder 24.51% (95% CI 18.16–31.46), and burnout 31.81% (95% CI 13.32–53.89) (38). However, in our study, despite high levels of depression, anxiety, post-traumatic stress, and burnout, only 18% of health workers and 11% of correctional officers reported mild to moderate sleep disturbance. Previous studies have found much higher rates of sleep disturbance in health care workers. For example, the same meta-analysis found the prevalence of sleeping difficulties at 39.88% (95% CI 27.70–52.72). The lower rates of sleep disturbance in the present study may reflect the different work environment in correctional settings, where typically 24-h shift schedules are followed, and sleep disturbance may be viewed as “normal.”

The participants were divided in two groups, those working in health care roles in correctional settings and correctional officers and administrators. Correctional administrators are front-line supervisors that share similar working conditions with correctional officers. There were significant differences in responses of these two groups. Specifically, in all but the burnout and posttraumatic stress domains, health care workers, on average, had higher scores on these measures than correctional officers. Thus, correctional health care workers appear to be at a particularly high risk of developing psychological distress during COVID-19.

Previous studies of health care workers in general hospital settings during COVID-19 and SARS have identified women/females at higher risk of increased stress (11, 13). However, in our sample, sex was only associated with GAD-7 scores on regression analysis and not with depression, sleep, post-traumatic stress, or burnout measures. Findings from previous studies on sex differences and burnout are rather mixed and at times contradictory. Some studies have indicated that women are more likely to report high levels of burnout (38), but others have not (39). Our finding of greater burnout in correctional officers is consistent with previous studies. For example, a recent study prior to COVID-19 comparing correctional officers and nurses also found greater job burnout in correctional officers (10).

Interestingly, we found a negative correlation with age and symptoms of depression, anxiety, and burnout. This may reflect the older workers being more likely to be in higher positions with more job security and less stressful work environments within correctional facilities. Previous studies have shown that lower work-related position is associated with a higher level of psychological symptoms (40, 41). It is also possible that younger workers are spending more time on social media, thus being exposed to misinformation about COVID-19, which may increase anxiety.

Increased workload and workplace conflict were associated with all mental health domains except sleep disturbance and resilience. This is not surprising in the prison environment where teamwork and work force cohesion can literally be a matter of life and death. Lack of coworker support has been linked to increased burnout in correctional settings (42). Correctional workers work in closed confined environments for long shifts, often without any cell phones, internet, or other forms of contact with the outside world and may rely even more on each other for support as compared to general hospital workers.

Surprisingly, we found that depression, anxiety, burnout, and post-traumatic stress scores were higher when workers believed that institutional isolation practices were sufficient at protecting them from COVID-19. Similar findings were reported in a previous study in correctional officers that showed that institutional trust did not have a significant association with feeling effective at work (43). A large population study during COVID-19 from New Zealand also showed that higher institutional trust was associated with higher psychological distress (42). This seems counter-intuitive as access to protective equipment should ease staff anxiety. However, in jail settings, it may mean that access to protective equipment is quite limited and the staff with protective equipment are assigned more stressful tasks such as visiting inmates in their cells. Similarly, staff who were afraid of falling ill with COVID-19, scored high on most measures of psychological distress and low on the resilience scale.

Our finding that only 11–18% of participants reported sleep disturbance is inconsistent with previous COVID-19 studies where rates have ranged from 20 to 34% (13, 14). However, these studies were done in general hospital settings. It is possible that the correctional staff are used to disrupted sleep due to long, sometimes 24-h shifts and therefore see it as part of their work environment.

Despite correctional workers experiencing significant mental health challenges, we found high rates of resilience both in health care workers and correctional officers (55 and 64%, respectively). This is an encouraging finding, since fostering resilience has been shown to be associated with a reduction in stress and improved job performance in many occupational groups (44). Our data also support the hypothesis that psychological distress is inversely correlated with resilience.

Contrary to the previous studies on healthcare workers during the pandemic, working in COVID-19-unit was not found to have a statistically significant relations in any of the regression models. However, it should be noted that none of the previous studies were carried out in correctional settings. This suggests that in the present sample, correctional workers' perceptions of risk were perhaps a more important driver of perceived mental health burden than actual work conditions. Also, in our sample correctional staff provided cross coverage and were rotated throughout the prison or jail. They did not stay at any specific unit for more than few days and this may be the reason why we did not find any correlation with working in COVID-19-unit.

To our knowledge, this is the first study surveying the mental health of correctional workers during the COVID-19 pandemic. Our findings suggest that correctional workers face high psychological morbidity amid unique challenges in the correctional environment. Nevertheless, this study has several limitations of a cross-sectional design. Although our study had a relatively high response rate, response bias may still exist if the non-respondents were either too stressed to respond or not interested as they were not stressed, about 80% of the participants were health care workers. This was likely due to the fact the correctional officers are government employees and needed departmental permission to participate in research, while health care workers were contracted employees through a 3rd party called PrimeCare medical, Inc. (which provides healthcare services to county jails, prisons, and juvenile detention centers throughout the Northeastern United States) and did not require such permission. This differential response may have biased the results, as the correctional officers who were able to get departmental clearance may have been more motivated to participate.

Correctional facilities can become pandemic hotspots and drive transmission in surrounding communities, thereby making correctional facilities an important focus of public health interventions. The well-being of correctional workers is a key factor in such interventions. Correctional populations and staff have a much lower rate of COVID-19 vaccination^3^ and high flow into and out of correctional facilities will continue to threaten those imprisoned, the staff, and the larger community (44–46). Further research and resources are required for long term planning and crisis response during pandemics to mitigate the stress and psychological impact on correctional workers (47).

## Author's Note

PrimeCare medical provides comprehensive health care services to county jails, prisons and juvenile detention centers throughout the Northeastern United States.

## Data Availability Statement

The original contributions presented in the study are included in the article/Supplementary Material, further inquiries can be directed to the corresponding author/s.

## Ethics Statement

The studies involving human participants were reviewed and approved by Solutions IRB. The patients/participants provided their written informed consent to participate in this study.

## Author Contributions

All authors listed have made a substantial, direct, and intellectual contribution to the work and approved it for publication.

## Conflict of Interest

PR-M and TW were employed by the company PrimeCare. The remaining authors declare that the research was conducted in the absence of any commercial or financial relationships that could be construed as a potential conflict of interest.

## Publisher's Note

All claims expressed in this article are solely those of the authors and do not necessarily represent those of their affiliated organizations, or those of the publisher, the editors and the reviewers. Any product that may be evaluated in this article, or claim that may be made by its manufacturer, is not guaranteed or endorsed by the publisher.

## Footnotes

^1^ https://www.themarshallproject.org/2020/12/18/1-in-5-prisoners-in-the-us-has-had-covid-19

^2^ https://coronavirus.jhu.edu/map.html

^3^ https://www.prisonpolicy.org/blog/2021/04/22/vaccinerefusal/
