# Supplementary material for: Mental Health of Staff at Correctional Facilities in the United States During the COVID-19 Pandemic
Source: Front Psychiatry. 2022 Jan 25;12:767385. doi: 10.3389/fpsyt.2021.767385 (PMC8821525; doi:10.3389/fpsyt.2021.767385)
Supplement: Supplementary file 1 [file Data_Sheet_1.pdf]

Appendix A

Regression Correlation Matrices

Table A1

Descriptive Statistics and Correlation Matrix for PHQ-9 Variables (n = 516)

Variable	*M*	*SD*	1	2	3	4	5	6	7	8	9	10
1. PHQ	5.52	5.08	-									
2. Protective Equipment [a]	2.78	1.43	**.29**	-								
3. COVID-19 Fear [b]	0.45	0.50	**.24**	**.23**	-							
4. Increased Workload [b]	0.58	0.50	**.30**	**.15**	**.11**	-						
5. Work Conflict [b]	0.49	0.50	**.29**	**.20**	**.13**	**.35**	-					
6. COVID-19 Unit Infection [b]	0.55	0.50	**.14**	**.13**	.03	**.20**	**-.20**	-				
7. Quarantined [b]	0.20	0.40	**.11**	.03	**.07**	**.07**	**.11**	**.15**	-			
8. Age [c]	2.80	0.87	**-.15**	**-.18**	.01	**-.08**	**-.06**	-.06	-.03	-		
9. Sex [d]	0.76	0.43	**.15**	**.21**	**.13**	**.10**	**.07**	**.08**	.00	**-.20**	-	
10. Self-Isolation [b]	0.15	0.36	**.17**	**.10**	**.14**	.07	**.13**	**.17**	**.37**	**-.09**	.02	-
11. Chronic Medical [b]	0.33	0.47	**.14**	**.04**	**.15**	.03	.05	**.08**	**.11**	**.10**	.04	[illegible]
12. Lives Alone [b]	0.07	0.26	.04	-.04	**-.08**	-.05	-.06	-.01	-.02	**.11**	.01	[illegible]
13. Work Position [e]	0.18	0.38	**-.14**	**-.18**	**-.19**	**-.14**	**-.08**	**-.13**	**-.04**	.06	**-.53**	[illegible]

Note: Bold values show significance at $p < .05$.

[a] 1 = Definitely yes, 2 = Probably yes, 3 = Might or might not, 4 = Probably not, 5 = Definitely not

[b] 0 = No, 1 = Yes

[c] 1 = 18-24, 2 = 25-34, 3 = 35-49, and 4 = 50-65

[d] 0 = Male, 1 = Female

[e] 0 = Health Care Worker, 1 = Correctional Officer

Table A2

Descriptive Statistics and Correlation Matrix for GAD7 Variables (n = 520)

Variable	*M*	*SD*	1	2	3	4	5	6	7	8	9	10
1. PHQ	6.05	5.79	-									
2. Protective Equipment [a]	2.77	1.42	**.26**	-								
3. COVID-19 Fear [b]	0.45	0.50	**.29**	**.24**	-							
4. Increased Workload [b]	0.58	0.49	**.30**	**.15**	**.11**	-						
5. Work Conflict [b]	0.49	0.50	**.30**	**.19**	**.14**	**.35**	-					
6. COVID-19 Unit Infection [b]	0.55	0.50	**.13**	**.14**	.03	**.18**	**.19**	-				
7. Quarantined [b]	0.21	0.40	**.14**	.04	**.09**	.07	**.09**	**.16**	-			
8. Age [c]	2.81	0.87	**-.25**	**-.19**	-.01	**-.10**	-.06	**-.07**	-.02	-		
9. Sex [d]	0.76	0.43	**.18**	**.21**	**.12**	**.09**	.06	**.08**	.01	**-.20**	-	
10. Self-Isolation [b]	0.16	0.37	**.15**	**.08**	**.14**	**.07**	**.12**	**.15**	**.37**	**-.09**	.04	-
11. Chronic Medical [b]	0.33	0.47	**.09**	.04	**.15**	.04	.05	.06	.10	**.09**	.04	**.1**[illegible]
12. Lives Alone [b]	0.07	0.26	-.05	-.04	**-.09**	-.06	-.05	-.01	**-.02**	**.11**	.00	.0[illegible]
13. Work Position [e]	0.18	0.38	**-.11**	**-.18**	**-.19**	**-.14**	**-.08**	**-.12**	**-.05**	.06	**-.53**	-.0[illegible]

Note: Bold values show significance at $p < .05$.

[a] 1 = Definitely yes, 2 = Probably yes, 3 = Might or might not, 4 = Probably not, 5 = Definitely not

[b] 0 = No, 1 = Yes

[c] 1 = 18-24, 2 = 25-34, 3 = 35-49, and 4 = 50-65

[d] 0 = Male, 1 = Female

[e] 0 = Health Care Worker, 1 = Correctional Officer

Table A3

Descriptive Statistics and Correlation Matrix for MBI-2 Variables (n = 525)

Variable	*M*	*SD*	1	2	3	4	5	6	7	8	9	10
1. MBI-2	3.76	3.05	-									
2. Protective Equipment [a]	2.76	1.43	**.33**	-								
3. COVID-19 Fear [b]	0.45	0.50	**.14**	**.24**	-							
4. Increased Workload [b]	0.58	0.49	**.37**	**.16**	**.16**	-						
5. Work Conflict [b]	0.49	0.50	**.38**	**.19**	**.13**	**.36**	-					
6. COVID-19 Unit Infection [b]	0.55	0.50	**.15**	**.14**	.04	**.18**	**.19**	-				
7. Quarantined [b]	0.21	0.41	**.10**	.03	**.08**	.06	**.09**	**.16**	-			
8. Age [c]	2.82	0.86	**-.18**	**-.19**	-.00	-.10	-.06	-.07	-.03	-		
9. Sex [d]	0.76	0.43	.03	**.20**	**.12**	**.09**	.05	**.07**	.00	**-.19**	-	
10. Self-Isolation [b]	0.16	0.37	.05	**.08**	**.14**	**.06**	**.11**	**.15**	**.37**	**-.09**	.04	-
11. Chronic Medical [b]	0.33	0.47	.03	.04	**.16**	.03	.04	.07	**.11**	**.10**	.03	**.13**
12. Lives Alone [b]	0.07	0.26	-.01	-.04	-.09	-.06	-.05	-.01	-.02	**.11**	.00	.02
13. Work Position [e]	0.17	0.38	**.11**	**-.19**	**-.18**	**-.13**	-.07	**-.12**	-.04	.05	**-.53**	-.0[illegible]

Note: Bold values show significance at $p < .05$.

[a] 1 = Definitely yes, 2 = Probably yes, 3 = Might or might not, 4 = Probably not, 5 = Definitely not

[b] 0 = No, 1 = Yes

[c] 1 = 18-24, 2 = 25-34, 3 = 35-49, and 4 = 50-65

[d] 0 = Male, 1 = Female

[e] 0 = Health Care Worker, 1 = Correctional Officer

Table A4

Descriptive Statistics and Correlation Matrix for PROMIS Sleep Variables (n = 523)

Variable	*M*	*SD*	1	2	3	4	5	6	7	8	9	1[illegible]
1. MBI	10.86	1.83	-									
2. Protective Equipment [a]	2.75	1.42	**.10**	-								
3. COVID-19 Fear [b]	0.44	0.50	**.08**	**.23**	-							
4. Increased Workload [b]	0.58	0.49	-.00	**.16**	**.12**	-						
5. Work Conflict [b]	0.49	0.50	.01	**.20**	**.14**	**.36**	-					
6. COVID-19 Unit Infection [b]	0.54	0.50	.07	**.13**	.04	**.19**	**.20**	-				
7. Quarantined [b]	0.21	0.40	.01	.03	**.09**	.06	**.10**	**.15**	-			
8. Age [c]	2.81	0.86	-.07	**-.19**	-.02	**-.09**	-.07	-.06	-.01	-		
9. Sex [d]	0.76	0.43	**.13**	**.20**	**.12**	**.09**	.06	**.08**	.00	**-.21**	-	
10. Self-Isolation [b]	0.16	0.37	.03	.09	**.15**	.07	**.12**	**.15**	**.37**	**-.08**	.04	
11. Chronic Medical [b]	0.33	0.47	.07	.04	**.15**	.04	.04	**.08**	**.12**	**.09**	.03	[illegible]
12. Lives Alone [b]	0.07	0.25	-.04	-.05	**-.09**	-.06	.07	.01	-.01	**.11**	.00	[illegible]
13. Work Position [e]	0.17	0.38	**.13**	**-.19**	**-.19**	**-.13**	**-.07**	**-.13**	-.03	.06	**-.54**	[illegible]

Note: Bold values show significance at *p* < .05.

[a] 1 = Definitely yes, 2 = Probably yes, 3 = Might or might not, 4 = Probably not, 5 = Definitely not

[b] 0 = No, 1 = Yes

[c] 1 = 18-24, 2 = 25-34, 3 = 35-49, and 4 = 50-65

[d] 0 = Male, 1 = Female

[e] 0 = Health Care Worker, 1 = Correctional Officer

Table A5

Descriptive Statistics and Correlation Matrix for IES-R Total Score Variables (n = 492)

Variable	*M*	*SD*	1	2	3	4	5	6	7	8	9
1. Constant	15.26	16.20	-								
2. Protective Equipment [a]	2.76	1.42	**.26**	-							
3. COVID-19 Fear [b]	0.44	0.50	**.29**	**.23**	-						
4. Increased Workload [b]	0.58	0.49	**.30**	**.16**	**.12**	-					
5. Work Conflict [b]	0.48	0.50	**.29**	**.19**	**.11**	**.37**	-				
6. COVID-19 Unit Infection [b]	0.54	0.50	**.18**	**.15**	.05	**.20**	**.20**	-			
7. Quarantined [b]	0.20	0.40	**.14**	.02	.07	.06	**.09**	**.17**	-		
8. Age [c]	2.81	0.87	**-.14**	**-.20**	-.04	**-.09**	-.07	-.07	-.02	-	
9. Sex [d]	0.77	0.42	.09	**.21**	**.12**	.07	.06	**.09**	-.02	**-.19**	-
10. Self-Isolation [b]	0.16	0.36	**.22**	.08	**.16**	.07	**.11**	**.14**	**.37**	**-.08**	.02
11. Chronic Medical [b]	0.33	0.47	**.14**	.02	**.16**	.04	.05	.07	**.12**	**.09**	.02
12. Lives Alone [b]	0.75	0.26	-.06	-.03	-.08	-.07	-.04	.00	-.01	**.12**	-.01
13. Work Position [e]	0.17	0.38	-.04	**.16**	**-.17**	**-.13**	-.05	**.14**	-.04	**.08**	**-.53**

Note: Bold values show significance at $p < .05$.

[a] 1 = Definitely yes, 2 = Probably yes, 3 = Might or might not, 4 = Probably not, 5 = Definitely not

[b] 0 = No, 1 = Yes

[c] 1 = 18-24, 2 = 25-34, 3 = 35-49, and 4 = 50-65

[d] 0 = Male, 1 = Female

[e] 0 = Health Care Worker, 1 = Correctional Officer

Table A6

Descriptive Statistics and Correlation Matrix for IES -R Avoidance Variables (n = 507)

Variable	*M*	*SD*	1	2	3	4	5	6	7	8	9
1. Constant	5.50	6.16	-								
2. Protective Equipment [a]	2.76	1.42	**.23**	-							
3. COVID-19 Fear [b]	0.44	0.50	**.23**	**.24**	-						
4. Increased Workload [b]	0.58	0.49	**.25**	**.16**	**.12**	-					
5. Work Conflict [b]	0.48	0.50	**.26**	**.18**	**.12**	**.37**	-				
6. COVID-19 Unit Infection [b]	0.54	0.50	**.16**	**.14**	.05	**.20**	**.20**	-			
7. Quarantined [b]	0.20	0.40	**.09**	.02	**.08**	.06	**.08**	**.16**	-		
8. Age [c]	2.80	0.87	-.13	**-.19**	-.03	**-.09**	**-.07**	-.07	-.01	-	
9. Sex [d]	0.77	0.42	.01	**.20**	**.12**	**.08**	.05	**.10**	-.01	**-.19**	-
10. Self-Isolation [b]	0.15	0.36	**.16**	**.08**	**.16**	**.07**	**.11**	**.14**	**.38**	**-.08**	.03
11. Chronic Medical [b]	0.33	0.47	**.11**	.03	**.16**	.05	.05	.07	**.12**	**.09**	.03
12. Lives Alone [b]	0.07	0.26	**-.08**	-.03	**-.08**	-.07	-.04	.00	-.01	**.12**	-.01
13. Work Position [e]	0.18	0.38	.03	**-.17**	**-.17**	**-.14**	-.06	**-.14**	-.05	**.07**	**-.53**

Note: Bold values show significance at $p < .05$.

[a] 1 = Definitely yes, 2 = Probably yes, 3 = Might or might not, 4 = Probably not, 5 = Definitely not

[b] 0 = No, 1 = Yes

[c] 1 = 18-24, 2 = 25-34, 3 = 35-49, and 4 = 50-65

[d] 0 = Male, 1 = Female

[e] 0 = Health Care Worker, 1 = Correctional Officer

Table A7

Descriptive Statistics and Correlation Matrix for IES-R Hyperarousal Variables (n = 518)

Variable	*M*	*SD*	1	2	3	4	5	6	7	8	9	
1. Constant	4.20	4.85	-									
2. Protective Equipment [a]	2.77	1.42	**.24**	-								
3. COVID-19 Fear [b]	0.45	0.50	**.29**	**.23**	-							
4. Increased Workload [b]	0.58	0.49	**.28**	**.16**	**.13**	-						
5. Work Conflict [b]	0.49	0.50	**.29**	**.18**	**.13**	**.36**	-					
6. COVID-19 Unit Infection [b]	0.54	0.50	**.14**	**.13**	.03	**.20**	**.19**	-				
7. Quarantined [b]	0.21	0.40	**.11**	.02	**.08**	.06	**.09**	**.16**	-			
8. Age [c]	2.81	0.87	**-.12**	**-.18**	-.02	**-.09**	-.06	**-.08**	-.02	-		
9. Sex [d]	0.76	0.43	**.12**	**.21**	**.13**	**.08**	.06	**.08**	-.01	**-.20**	-	
10. Self-Isolation [b]	0.16	0.37	**.17**	**.08**	**.15**	.06	.11	**.15**	**.37**	**-.08**	.03	
11. Chronic Medical [b]	0.34	0.47	**.15**	.04	**.16**	.04	**.05**	.07	**.11**	**.10**	.02	[illegible]
12. Lives Alone [b]	0.71	0.26	-.06	-.03	**-.08**	-.07	-.05	.00	-.01	**.11**	.00	
13. Work Position [e]	0.17	0.38	-.08	**-.18**	**-.19**	**-.12**	-.06	**-.12**	-.03	.06	**-.54**	[illegible]

Note: Bold values show significance at $p < .05$.

[a] 1 = Definitely yes, 2 = Probably yes, 3 = Might or might not, 4 = Probably not, 5 = Definitely not

[b] 0 = No, 1 = Yes

[c] 1 = 18-24, 2 = 25-34, 3 = 35-49, and 4 = 50-65

[d] 0 = Male, 1 = Female

[e] 0 = Health Care Worker, 1 = Correctional Officer

Table A8

Descriptive Statistics and Correlation Matrix for IES-R Intrusion Variables (n = 515)

Variable	*M*	*SD*	1	2	3	4	5	6	7	8	9	
1. Constant	5.66	6.41	-									
2. Protective Equipment [a]	2.76	1.42	**.25**	-								
3. COVID-19 Fear [b]	0.44	0.50	**.32**	**.23**	-							
4. Increased Workload [b]	0.58	0.49	**.28**	**.16**	**.13**	-						
5. Work Conflict [b]	0.48	0.50	**.28**	**.19**	**.12**	**.37**	-					
6. COVID-19 Unit Infection [b]	0.53	0.50	**.16**	**.13**	.04	**.19**	**.19**	-				
7. Quarantined [b]	0.21	0.40	**.17**	.03	**.08**	.05	**.09**	**.16**	-			
8. Age [c]	2.80	0.87	**-.11**	**-.19**	-.02	**-.09**	**-.08**	-.07	-.02	-		
9. Sex [d]	0.77	0.42	**.12**	**.21**	**.13**	**.08**	.07	**.09**	-.01	**-.19**	-	
10. Self-Isolation [b]	0.16	0.36	**.23**	**.09**	**.15**	.06	**.12**	**.16**	**.37**	**-.09**	.04	
11. Chronic Medical [b]	0.33	0.47	**.13**	.03	**.15**	.04	.04	.07	**.11**	**.09**	.03	[illegible]
12. Lives Alone [b]	0.07	0.26	-.03	-.03	-.08	-.07	-.04	.00	-.01	**.12**	-.01	
13. Work Position [e]	0.18	0.38	**-.12**	**-.18**	**-.18**	**-.14**	-.07	**-.13**	-.04	.07	**-.54**	[illegible]

Note: Bold values show significance at $p < .05$.

[a] 1 = Definitely yes, 2 = Probably yes, 3 = Might or might not, 4 = Probably not, 5 = Definitely not

[b] 0 = No, 1 = Yes

[c] 1 = 18-24, 2 = 25-34, 3 = 35-49, and 4 = 50-65

[d] 0 = Male, 1 = Female

[e] 0 = Health Care Worker, 1 = Correctional Officer

Table A9

Descriptive Statistics and Correlation Matrix for CD-RISC-2 Variables (n = 526)

Variable	*M*	*SD*	1	2	3	4	5	6	7	8	9	
1. Constant	6.61	1.57	-									
2. Protective Equipment [a]	2.77	1.42	**-.17**	-								
3. COVID-19 Fear [b]	0.45	.50	**-.24**	**.23**	-							
4. Increased Workload [b]	0.58	0.50	-.04	**.16**	**.12**	-						
5. Work Conflict [b]	0.49	0.50	-.05	**.19**	**.13**	**.36**	-					
6. COVID-19 Unit Infection [b]	0.55	0.50	.00	**.14**	.04	**.18**	**.19**	-				
7. Quarantined [b]	0.21	0.40	**-.08**	.03	**.08**	.06	**.09**	**.15**	-			
8. Age [c]	2.81	0.87	.06	**-.18**	-.01	**-.09**	-.07	-.07	-.02	-		
9. Sex [d]	0.76	0.43	**-.16**	**.20**	**.12**	**.09**	.06	**.08**	.01	**-.20**	-	
10. Self-Isolation [b]	0.16	0.36	-.01	**.09**	**.15**	.06	**.12**	**.15**	**.37**	**-.09**	.04	
11. Chronic Medical [b]	0.34	0.47	-.07	.04	**.15**	.03	.05	.07	**.11**	**.10**	.03	[illegible]
12. Lives Alone [b]	0.07	0.26	-.05	-.03	**-.08**	-.07	-.05	.00	-.01	**.11**	.00	
13. Work Position [e]	0.17	0.38	**-.07**	**-.18**	**-.18**	**-.14**	**-.07**	**-.13**	-.05	.06	**-.52**	[illegible]

Note: Bold values show significance at *p* < .05.

[a] 1 = Definitely yes, 2 = Probably yes, 3 = Might or might not, 4 = Probably not, 5 = Definitely not

[b] 0 = No, 1 = Yes

[c] 1 = 18-24, 2 = 25-34, 3 = 35-49, and 4 = 50-65

[d] 0 = Male, 1 = Female

[e] 0 = Health Care Worker, 1 = Correctional Officer
